# Reconstruction of post burn scalp alopecia by using expanded hair-bearing scalp flaps

**DOI:** 10.12669/pjms.316.7927

**Published:** 2015

**Authors:** Farhat ul ann Tayyaba, Mohammad Mughees Amin, Sohail Attaur-Rasool, Uzma Naseer, Akashah Ambar

**Affiliations:** 1 Dr. Farhat-ul-Ann Tayyaba, FCPS. Department of Plastic and Reconstructive Surgery, Bahawal Victoria Hospital, Bahawalpur, Pakistan; 2 Dr. Mohammad Mughees Amin, FCPS. Department of Plastic and Reconstructive Surgery, Bahawal Victoria Hospital, Bahawalpur, Pakistan; 3 Dr. Sohail Attaur-Rasool, Department of Physiology, Quaid-e-Azam Medical College, Bahawalpur; 4 Dr. Uzma Naseer, FCPS. Department of Plastic and Reconstructive Surgery, Bahawal Victoria Hospital, Bahawalpur, Pakistan; 5 Dr. Akashah Ambar, MBBS. Department of Plastic and Reconstructive Surgery, Bahawal Victoria Hospital, Bahawalpur, Pakistan

**Keywords:** Tissue expansion, Scalp reconstruction, Burn

## Abstract

**Background and Objective:**

Tissue expansion is a time-tested and frequently used procedure for utilizing local tissue to replace large defects. We aimed to assess the success & complications of tissue expansion in correction of post burn scalp alopecia.

**Methods:**

In this study, 30 patients of scalp burn alopecia of 5 to 35 years age group were treated with tissue expansion of the scalp at Bahawal-Victoria Hospital from January 2013 to December 2014. The area of the scalp loss was within 1/5 to 2/5. Our technique employed an insertion site distal to the area needed to be expanded, attempting to minimize complication like extrusion & wound dehiscence. The patients were followed-up weekly during first month and then fortnightly for next four months.

**Result:**

Our study involved 8 male (26.67%) and 22 female subjects (73.33%) with a mean age of 21years. Flame burn accounted for the mostly 53.3% (n=16) of scalp burns & parieto-temporal region was most commonly affected in 33.4% (n=10) of subjects. Desired aesthetic results were achieved in all the patients without any major complication. Minor complication included mild infection in 8 (26.67%), seroma in 4 (13.33%) & wound dehiscence in 2 (6.67%) patients.

**Conclusion:**

Tissue expansion is a simple, safe, & efficient technique for aesthetic scalp reconstruction. With a simple modification of distal incision and tunneling, we succeeded in minimizing complications. Versatile design of the expanded scalp flap can distribute the expanded hair-bearing scalp properly in the reconstructed recipient site.

## INTRODUCTION

Scalp is a specialized area of skin which covers the skull. Post-burn alopecia is a common condition requiring treatment of large areas of defects in skin and soft tissue which poses a real challenge to the reconstructive surgeon.^1^ The psychological impact of such an extensive defect is often quite traumatic for the patient. Hence, the reconstruction process is a difficult task for the surgical team in terms of both outcome of surgery and patient satisfaction. The conventional methods of rotational scalp flaps, serial excision, micro-vascular flap transfer and follicular unit transplantation have many drawbacks including lengthy hospital stays, flap necrosis and inability to cover major scalp defects.^2^ Moreover, Use of skin grafts or flaps from remote parts of the body may be cosmetically unacceptable because of bald spots on the scalp or mismatch of the colour.^3^

Tissue expansion is a technique in reconstructive surgery for ‘growing’ extra skin in an area of tissue loss due to burn, trauma or disease.^3^ Since described by Neumann in 1957, the procedure has undergone many advances.^4^ Currently, tissue expansion is accepted as a routine procedure in plastic and restorative surgery for the reconstruction of various defects and deformities. Since no other tissue can emulate functions of the human scalp adequately; tissue expansion has been the mainstay of treatment when hair-bearing scalp is lost to burns, traumatic avulsions, tumor resections, congenital anomalies and infections. The technique has been successfully utilized in post-burn alopecia of scalp.^5^-^7^ Its value in reconstruction of the scalp is based on the fact that the ‘new skin’ contains hair follicles as well as bears the same skin tone.

The purpose of this study was to evaluate the success and complications of tissue expansion in the correction of post burn scalp alopecia. Our technique employed an insertion site distal to the area needed to be expanded, attempting to minimize complication like extrusion & wound dehiscence.

## METHODS

This descriptive study was conducted from January 2013 to December 2014 in the Department of Plastic Surgery, Bahawal-Victoria Hospital, Bahawalpur. Thirty patients with scalp burn alopecia of age 5 to 35 years of either gender with stable scar were included in this study. Patients with active infections, open wound and diabetes mellitus were excluded. The study was approved by the ethical review committee of Bahawal-Victoria Hospital Bahawalpur and a written informed consent was obtained from all patients before inclusion.

The patient evaluation included personal and demographic data, mode of the scalp burn, duration of burn, and any medical or surgical treatment received. The surgical procedure was explained to the patients along with the possible outcomes and complications of the procedure in detail. The queries raised by the patients were also addressed to their satisfaction. We also ensured that the patients express their expectations regarding the outcome of the procedure in light of the information provided to them by the surgical team. Satisfied patients were offered enrolment in the study and the written consent was obtained. Preoperative photographs were captured, along with measurement of defect size and assessment of hair-bearing scalp for donor selection. If the size of the defect exceeded 25% of the total hair bearing scalp, it was decided to use multiple tissue expanders. Tissue expanders have silicone outer shells and employ remote valve system to allow for saline fluid injections and are available in different shapes (rectangular, rounded, and elliptical). Our technique involved use of a distal incision for insertion of the expander.

With the patient under general anesthesia, the expander was placed through a small incision in the healthy scalp beyond the donor area by creating a pocket between the galea aponeurotica and the pericranium. Incision for insertion for expander was always radial to the expander. Maximum attention was paid to ensure that the pockets were large enough to accommodate the expander in order to avoid ‘knuckles’ or ‘bends’ in the prostheses’ surfaces. We placed close suction drain in the pocket till all excess drainage stopped. Due to the distal site of incision, expansion was started next post-operative day with repeated over-filling technique using normal saline twice a week until the desired volume was obtained. Inflation was continued until the area of the expanded scalp attained at least 2.5 times the area of alopecia area to be reconstructed. In most patients, the desired results were obtained in 9 to 12 weeks.

The second stage consisted of the removal of the tissue expander and reconstruction of the defect. The flaps were planned before removing the tissue expander considering the size and shape of the defect and the quality and quantity of the expanded scalp. During designing of the expanded scalp flap, we ensured matching of the hair direction with the recipient site, especially in the reconstruction of the frontal hairline or ‘sideburn’. When the hair direction of the expanded area was parallel to the adjacent defect or situated in the posterior region, a simple advancement flap corrected alopecia adequately. However, if the hair direction of the expanded area was too much angular to the adjacent defect, then a rotation flap or rotation-advancement flap was preferred. We performed capsulectomy by cutting the surrounding capsule of the expander base in order to encourage mobility of the expanded tissue without problem of flap survival. All the data were entered in SPSS version 16 and analyzed accordingly. The Mean ± SD were calculated for numerical variables as well as frequencies and percentages were calculated for categorical variables.

**Case 1:**
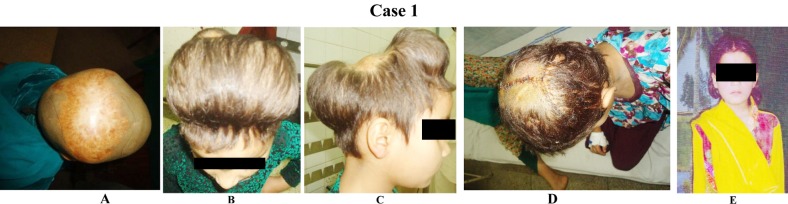
(A) 5-year-old girl presented with unstable post grafting scar in the right and left temporoparietal region following a burn. (B) The remaining scalp was expanded with two rectangular tissue expanders and the defects were repaired with the advancement flaps. (C)Lateral view of same patient.(D)Early postoperative view.(E) Late post-operative results after 6 months.

## RESULTS

Mean (±SD) age of the patients was 21.13 (±10.06) years. Among the total 30 patients, 8 (26.67%) were male and 22(73.33%) were female with male to female ratio of 1:1.7 The most common etiology of scalp burn was flame burn in 16 (53.3%) patients followed by chemical burn in 8 (26.7%) and scalds in 6 (20%)patients. The parieto-temporal region was involved in 10 patients (33.4%) followed by involvement of frontal region in 8 (26.7%), occipital and fronto-perital in 4(13.3%), occipito-temporal and temporal in 2 (6.7%) patients (Table-I). According to the shape of affected area, different shapes of expanders were utilized. Most commonly, rectangular shaped expanders were needed in 28 (66.67%) patients, round shaped in 8 (19.04%) patients and crescent shape expenders was used in 6 (14.26%) patients. Additionally, rectangular and round shape expanders were used in 4 (13.3%) patients. (Table-II).

**Table-I:** Sites of Scalp Reconstructed (n=30).

Site	Number	Percentage
Parieto-Temporal	10	33.4
Frontal	8	26.7
Occipital	4	13.3
Fronto-Parietal	4	13.3
Occipito-Temporal	2	6.7
Temporal	2	6.7
Total	30	100

**Table-II:** Shapes and numbers of expanders used in reconstruction of scalp burn alopecia (n=30).

Shape	Number	Percentage
Rectangular	28	66.67
Round	8	19.04
Crescent	6	14.26
Total	42	100

We achieved satisfactory aesthetic results in all patients. In 26 patients, total reconstruction of scalp alopecia was done; and in 4 patients where alopecia was extensive, we achieved aesthetic results by reconstructing frontal hair line and side burns. Majority16 (53.33%) of the patients had no post-operative compilations. Mild infection was observed and treated successfully in 26.67% patients. Seroma developed in 4 (13.33%) of our patients and wound dehiscence resulted in 2 (6.67%) patients. Both conditions were managed successfully (Table-III).

**Table-III:** Frequency of complications during reconstruction of scalp burn alopecia (n=30).

	Number	Percentage
No complication	16	53.33
Mild infection	8	26.67
Seroma	4	13.33
Wound dehiscence	2	6.67
Total	30	100

## DISCUSSION

Patients with burns of scalp usually present to reconstructive and plastic surgery for treatment of scalp alopecia. Serial excisions and a variety of local scalp flaps are usually successful in correcting small alopecia defects. However, scalp defects of more than 3 to 5cm width are usually challenging to correct because of the tensile force on the wound closure as well as due to phenomenon of ‘stretch-back’ which occurres subsequently.^7^ Hair grafting can be an option in treating patients presenting with large scalp defects, but it is usually difficult to achieve aesthetically natural-looking hair. Additionally, problems such as unstable scar and thin skin grafting are expected to cause break down, bleeding, or infection after attempted hair grafting resulting in no hair growth.^8^ Advent of tissue expansion has revolutionized the method of aesthetic reconstruction following scalp alopecia secondary to burns. The rich blood circulation, thick overlying tissue, and the enforced base make the scalp an ideal site for tissue expansion. Most importantly, the tissue expansion provides a natural hair bearing skin for scalp reconstruction with a near-normal hair density.^9^ The main issue of use of expander for reconstruction is high cost. But in our setup tissue expander were provided by government on regular basis.

**Case 2:**
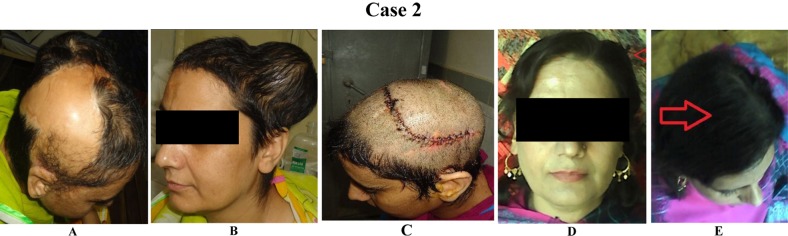
(A)This 34-year-old woman presented with a large area of unstable scar extending from the parietal to the occipital region following a burn.(B) opposite side was expanded with single round tissue expanders.(C) The defect was repaired using advancement flaps of expended skin. Early postoperative result.(D& E) Late post operative results after 6 months.

**Case 3:**
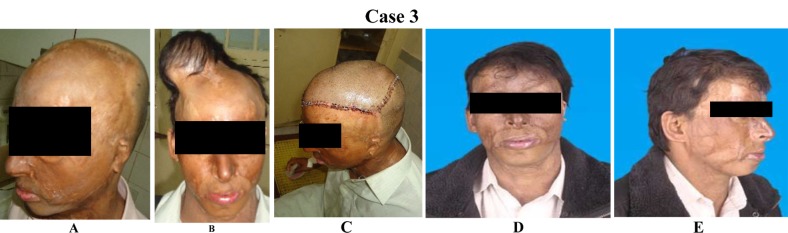
(A)An alopecia of fronto temporal scalp after chemical burn. (B)Remainingscalp expended by round expender. Fully expanded views. (C)Reconstruction by using an expanded advancement flap. Immediate postoperative view. (D) Late post-operative results after 6 months (frontal view). (E) Lateral view of same patient.

**Case 4:**
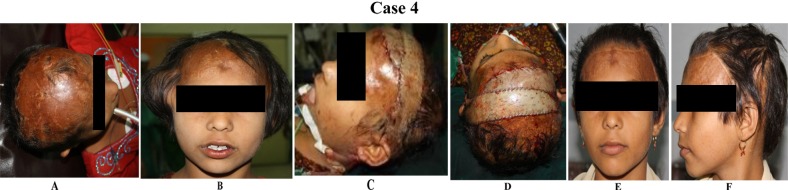
(A) This 6-year-old girl presented with a large area of unstable scar extending from the frontal to the parietal region following a burn.(B) Bilateral temporoparietal scalp is expanded with two rectangular tissue expanders. (C& D) The defect was repaired using two advancement flaps from the temporal area. Immediate post-operative results.(E & F) Late postoperative result. Note the corrected anterior hairline.

The selection of the flap usually depends on the characteristics of the patient and the surgeon’s preference. This decision on selection of the flap should be made before placement of the tissue expander in order to avoid unnecessary tissue loss. Major tissue loss during flap elevation has been reported for the advancement flaps, but can be eliminated with strategic addition of Z-plasty. The Z-plasties provide better adaptation of the expanded flap, enabling the surgeon to create a proper hairline and sideburns avoiding linear scar formation. On the other hand, the amount of expanded tissue needed to close the same size defect is more than needed for a rotation flap. We used slightly larger expanders to cover the defect especially for rectangular-shaped expanders. The advancement flaps provide the advantages of their simple design, easier technique, and fewer circulatory problems, even when the flaps were not elevated in an axial design.^10^

The results of our study have shown that using tissue expansion, total reconstruction of post-burn alopecia could be achieved in 90% of the treated patients. Our results were higher than that achieved by Hudson DA et al who had completely reconstructed 80% of his patients, and Yuosif Salih who reported 87.13% success rate. Moreover, our results are comparable to those achieved by Hafezi et al.^11^-^13^ The complication rate in our study was 46.67% including mild infection in 26.67% patients, seroma in 13.33% patients and wound dehiscence in 6.67% patients. The complication rate of Fochtmann et al. was52% which is comparable with our findings.^14^ Comparable postoperative complication rates were also observed by Cunha et al. as 51.4%.^15^ Lower post-operative complications were reported by DA Hudson (25%), Yousaf Salih (34.75%) and Farhad et al.(27%).^11^-^13^

In order to achieve full advantages and success of the procedure, the expansion process must also aim to minimize complication. The major complication in this procedure is expander extrusion which was fortunately not seen in any of our patients. We believed that the main reason for expander extrusion is inappropriate surgical technique. Prevention from implant exposure requires careful planning and certain modifications in surgical technique. We managed to prevent extrusion of our expanders by placing expanders in an adequate pocket and by placing incision far away from the area needed to be expanded.^16^ By this distant placed incision, we were able to achieve the extra advantage of early start of expansion procedure. It also seems to reduce the rate of complications that would occur later due to dehiscence of scar later extrusion of expander since the migration or recruitment of tissue during expansion causes the tissue to move toward expander. Furthermore as already mentioned in methods that our incision of insertion of expander was always radial which means they lied in the line drawn from the approximately the midpoint of the implant.^17^ In this way the incisions are not prone to being forced open when the expansion is started. By using these maneuvers our implant extrusion rate was zero. To lower the infection rate in tissue expansion, we ensured the proper aseptic environment with per-operative I/V cefezolin at the time of induction of anesthesia. We continued post-op same antibiotic till the removal of drain. Moreover, before placement of expander we irrigated the pocket with gentacin solution as it is abroad spectrum antibiotic which has good coverage against gram positive and important gram negative organisms responsible for expander infection. Our policy of placing close suction drain prevented further formation of seroma that leads to infection. We also concentrated on prevention of late infection by inflation of expander under sterile condition by using povidine-iodine solution for preparation of inflation site. Hence, tissue expanders provide beneficial effects for the reconstruction of post burn alopecia. Despite the disadvantage of being (at least) a two-stage procedure, the expander technique provides tissue of the same texture and color with minimal donor site morbidity.

## CONCLUSION

Our results have demonstrated that tissue expansion is a simple, safe, and efficient technique for aesthetic scalp reconstruction. With this simple modification of distal incision and tunneling, complications can be minimized. Versatile design of the expanded scalp flap can distribute the expanded hair-bearing scalp properly in the reconstructed recipient site.
